# Prognostic analysis and identification of M7G immune-related genes in lung squamous cell carcinoma

**DOI:** 10.3389/fimmu.2025.1515838

**Published:** 2025-03-03

**Authors:** Li Wenhui, Wu Nan, Han Jiayi, Xu Ye, He Chunyu, Li Zhongzhou, Lei Hongtao, Tian Hui

**Affiliations:** ^1^ Department of Radiation Oncology, The First Affiliated Hospital of Kunming Medical University, Kunming, China; ^2^ Department of Radiation Oncology, The Second Affiliated Hospital of Harbin Medical University, Harbin, China; ^3^ Respiratory Intensive Care Unit, The First Affiliated Hospital of Kunming Medical University, Kunming, China; ^4^ Respiratory Intensive Care Unit, The First Affiliated Hospital of Zhengzhou University, Zhengzhou, China; ^5^ Department of Operations Management, The First Affiliated Hospital of Kunming Medical University, Kunming, China; ^6^ Institute of Medicine and Nursing, Hubei University of Medicine, Shiyan, China; ^7^ School of Public Health, Kunming Medical University, Kunming, China

**Keywords:** m7G, lung squamous cell carcinomas (LUSC), immune-related genes, prognostic, risk model

## Abstract

**Background:**

In recent years, the clinical application of targeted therapies and immunotherapy has significantly improved survival outcomes for patients with lung adenocarcinomas(LUAD). However, due to fewer mutations, lung squamous cell carcinomas(LUSC) shows limited efficacy with targeted and immunotherapy, resulting in a notably lower 5-year survival rate compared to lung adenocarcinoma. The m7G modification plays an important role in tumorigenesis, progression, immune evasion, and therapeutic response. This study aims to develop a novel scoring system based on m7G modification and immune status to clinically predict the prognosis of patients with LUSC and to provide new therapeutic targets.

**Methods:**

In this study, we utilized RNA-seq data from the TCGA-LUSC database as the training set and GSE50081 from the GEO database as the validation set. Immunotherapy data were obtained from the IMMPORT database, and m7G data from previous research. Using bioinformatics, we developed a prognostic model for LUSC based on m7G pathway-related immune gene characteristics. We analyzed the correlation between the prognostic model and clinical pathological features of LUSC, as well as the model’s independent prognostic capability. Subsequently, patients were divided into high-risk and low-risk groups, and we examined the differences in enriched pathways, immune cell infiltration correlations, and drug sensitivity between the two groups.

**Results:**

The m7G immune-related genes FGA, CSF3R, and ORM1 increase the survival risk in patients with lung squamous cell carcinoma, whereas NTS exerts a protective effect. The prognostic risk model for lung squamous cell carcinoma (LUSC) based on m7G immune-related gene expression demonstrates that the overall survival of the high-risk group is significantly poorer than that of the low-risk group.

**Conclusion:**

The risk model developed based on m7G immune-related genes can help predict the clinical prognosis of LUSC patients and guide treatment decisions.

## 1 Introduction

N(7)-methylguanosine (m7G) is one of the crucial post-transcriptional modifications of messenger RNA (mRNA) found in both prokaryotes and eukaryotes, It binds to the 5h end of mRNA in a co-transcriptional manner during transcription initiation. m7G can be regulated throughout the mRNA life cycle to protect against exonuclease-mediated degradation (1–3). Growing evidence suggests that RNA modifications play a crucial role in lung cancer progression (4, 5). Epigenetic alterations in RNA and histones have been extensively studied in tumor progression, leading to the development of a variety of therapeutic modalities, including histone deacetylase inhibitors and drugs targeting hypoxia-related pathways (6). RNA modifications, including N6-methyladenosine (m6A), 5-methylcytidine (m5C), N1-methyladenosine (m1A), and m7G, have been found to play important roles in cellular differentiation, protein production, and biological regulation (7). Lung cancer development and progression depend not only on genetic variation but also on epigenetic dysregulation (8, 9). As an important component of epigenetic modifications, RNA modifications are involved in the regulation of numerous physiological processes and the occurrence of diseases (10). Dynamic regulation and disruption of these RNA modifications are also associated with the lung cancer development, maintenance and progression of lung cancer (11, 12).

In addition, RNA dynamic modifications may affect the functional response and maturation of tumor immune cells (13). It has been gradually recognized that tumorigenesis and development are not only related to the intrinsic genetic background of cancer cells, but also depend on the interaction between the tumor and various systems in the body, particularly the immune system (14, 15). m7G-related genes are thought to interact with immune pathways, potentially affecting the tumor microenvironment and the immune system’s ability to respond to malignancies (16), Especially with the development of monoclonal antibody drugs and immunotherapy, tumor immunity has become a hot spot in tumor research (17). To date, the relationship between the screening of immune-related molecules, their expression in LUSC and their impact on the prognosis of LUSC has not been thoroughly studied.

However, the relationship between m7G methylation and immune-related cells and factors has been less studied. A comprehensive analysis of this relationship could help to better understand the relationship between m7G-related isoforms and the immune system and reveal potential mechanisms that affect the prognosis of LUSC, providing new insights for treatment. Therefore, in this study, we identified m7G-related genes through the analysis of genes involved in the m7G pathway using consensus clustering and differential expression analysis. Subsequently, we intersected these genes with known immune-related genes to construct a prognostic risk model for lung squamous cell carcinoma (LUSC). This model may enhance the accuracy of prognostic assessment and provide novel therapeutic targets for LUSC patients.

## 2 Materials and methods

### 2.1 Data source

**Figure 1:**
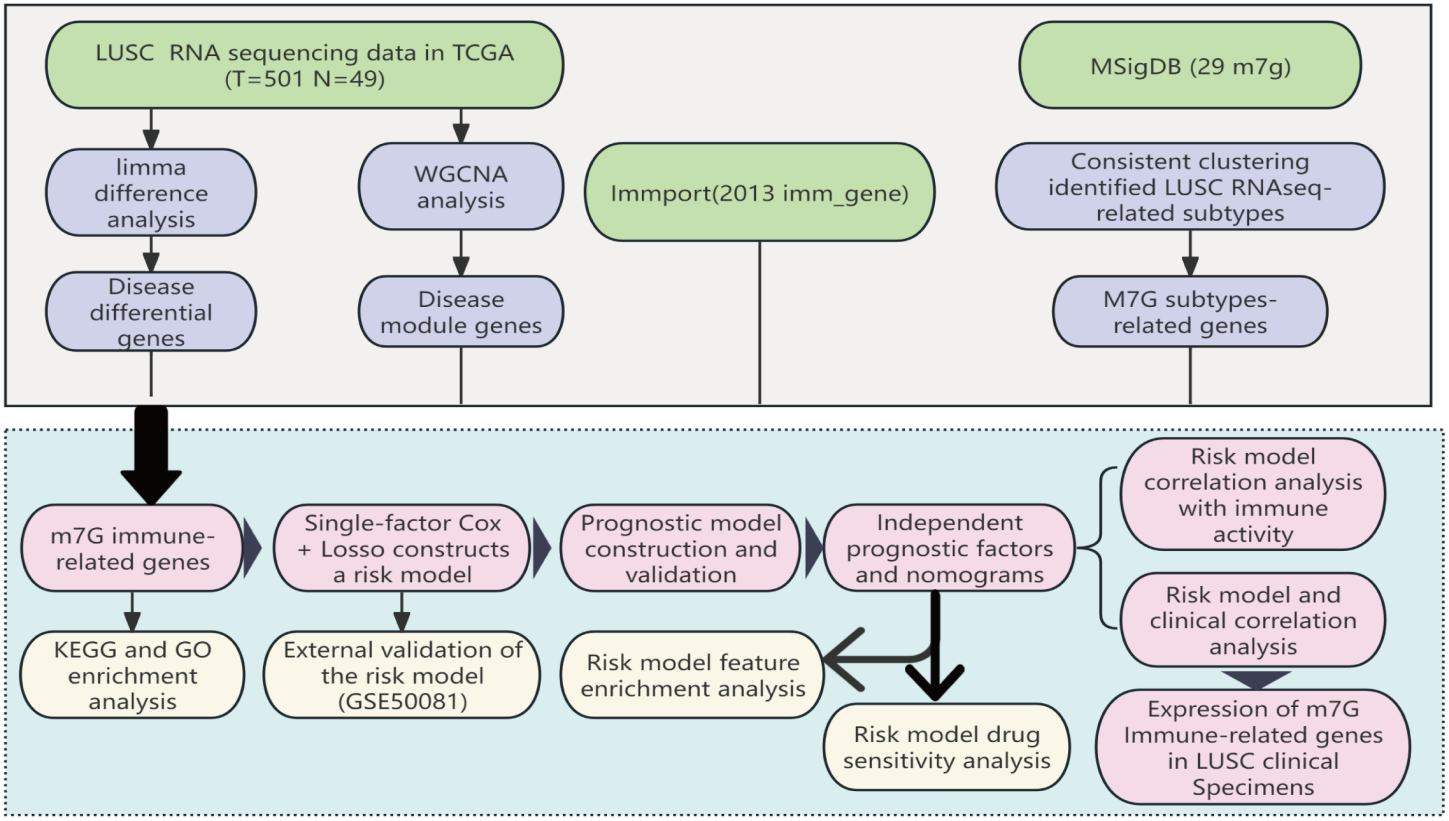
The workflow of the study: Based on data from the TCGA database, we identified differentially expressed m7G immune-related genes in LUSC and developed a prognostic model for these differential genes through LASSO-Cox regression analysis. This model has undergone various validations, proving to be stable and reliable. Based on this model, we further conducted Disease Ontology (DO) enrichment analysis, Gene Set Enrichment Analysis (GSEA), immune-related analysis, and drug sensitivity analysis to determine the potential functions of the prognostic markers.

RNA-seq (database updated to July 8, 2019) data for TCGA-LUSC were downloaded from the TCGA (https://portal.gdc.cancer.gov) database, including gene names, sample numbers, and expression data, and clinical data including patient IDs, survival time, survival status, age, sex, tumor grade, and TNM staging. RNA sequencing data and associated clinical characteristics of 550 LUSC patients were extracted from The Cancer Genome Atlas (TCGA) database, including 49 normal tissues and 501 LUSC tissues, were used for normal and cancer differential expression analysis. We utilized 501 patient samples for modeling analysis. Additionally, the GSE50081 dataset (55 patients with squamous cell carcinoma of the lung with survival information) dataset from the GEO (https://www.ncbi.nlm.nih.gov/geo/) database was downloaded for differential validation. 2013 immune-related genes were downloaded from the IMMPORT database in this study.)M7G-related genes: were obtained from the published review of Zhouhua Li (2022) (18), The workflow of the study is displayed in **Figure 1**.

### 2.2 Differential gene expression analysis of lung squamous carcinoma disease

#### 2.2.1 The limma software package was used to analyze mRNA data comprising 49 control samples and 501 disease samples

Significant mRNA differences were identified using the criteria of P_value<0.05 and |Log2FC|>0.5 to screen for disease-associated genes.

#### 2.2.2 Weighted co-expression analysis of mRNAs from LUSC was conducted using the R package “WGCNA”

Significant modules in the LUSC transcriptome data were identified using a threshold of β=5 (i.e., power value, combined with the R^2 value of the network and the degree of connectivity of the network), and from these modules, those showing the highest absolute values of correlation coefficients with the clinical traits were filtered and selected as the key modules for the subsequent analyses.

### 2.3 Differential module m7G immune gene screening and target gene function enrichment

Consensus clustering analysis was performed using the R package “ConsensusClusterPlus R” based on the expression of m7G-related genes to classify LUSC samples into different molecular subtypes (19). To investigate the clinical value of m7G-based subtypes, clinicopathologic features including age, gender, and staging were analyzed between subtypes. In addition, differences in OS between subtypes were explored using the R package. Genes associated with the m7G subtype were analyzed using the limma software package, with differences screened for P <0.05 and |Log2FC|>0.5. Finally, Key m7G immune-related genes were obtained using the intersection of disease and normal differential genes, modular genes, and m7G-related genes with 2013 immune genes in the Immport database (20). KEGG and GO enrichment analyses were performed using the clusterProfiler package in R, aiming to identify functions and associated pathways prevalent among a significant number of genes within a target gene set. Statistical methods were employed to accumulate hypergeometric distributions, employed the analysis whether a set of genes over- represented at a specific functional node (over-presentation). The specific formula for its calculation is as follows:


$$\text{p}\left(\text{X}>\text{q}\right)=1-\sum_{\text{x}=1}^{\text{q}}\frac{\left(\begin{array}{c} \text{n}\\ \text{x} \end{array}\right)\left(\begin{array}{c} \text{N}-\text{n}\\ \text{M}-\text{x} \end{array}\right)}{\left(\begin{array}{c} \text{N}\\ \text{M} \end{array}\right)}$$

### 2.4 Construction and validation of a risk model for M7G-associated immunity genes

The expression data of M7G-related immune genes (FPKM) were obtained in the previous step, and the TCGA-LUSC dataset was divided into a training set and a validation set in a ratio of 7:3, with 346 samples in the training set and 147 samples in the validation set. Univariate Cox and Lasso Cox regression analyses were performed, and prognostic risk models were constructed using the “cph” function of the R software “survival” package.

The formula for calculating the risk score is:


RiskScore =∑(βi×Xi)

Where indicates the coefficient of each gene in the multifactorial Cox regression analysis, and X indicates gene expression. Using and the median risk score value as the boundary, the patients were divided into high-risk and low-risk groups. Kaplan-Meier survival analysis was employed used to compare the prognosis of high-risk and low-risk groups, and ROC curves were plotted and AUC values were calculated to evaluate the predictive efficacy of the model.

To validate the accuracy of the risk model, the external GSE50081 dataset was used to merge the risk score with a LUSC dataset file that included clinicopathological factors such as T-stage, N-stage, age, clinical stage, and risk score to assess the correlation of the risk model with clinical features.

### 2.5 Characterization of m7G immune-related gene risk model and construction of column line diagrams

Univariate Cox regression analysis was used to identify clinical factors affecting prognosis. These factors were then included in multifactorial Cox regression analysis to screen for independent risk factors impacting the prognosis of LUSC. The independent prognostic model of clinical factors was constructed using the “cph” function from the R “rms” package. A nomogram visualizing this predictive model was constructed to predict the probable 1-, 3-, and 5-year survival rates of patients with LUSC was constructed. The predictive accuracy of the nomograms was evaluated using calibration curves, also implemented via the “rms” package in R.

### 2.6 Immune cell infiltration, immunoassay site analysis and drug sensitivity analysis

Immune cell infiltration analysis was performed using a variety of bioinformatics methods, including GSEA enrichment analysis, ESTIMATE, immune checkpoint analysis, drug sensitivity analysis, and single sample gene set enrichment analysis (ssGSEA) (21, 22), The ESTIMATE algorithm was used to infer the proportion of stromal cells and immune cells in a tumor sample based on gene expression. Subsequently, the ssGSEA algorithm was applied to evaluate immune cells populations, immune function, and the activity of immune pathways in each sample. By grouping samples based on immune activity, we could study the differences in immune function between high- and low risk-groups. Additionally, drug sensitivity was assessed in each group using the “pRRophtic” software package (23). The half-maximal inhibitory concentrations (IC50) of the drugs in the high-and low-split groups were compared by the Wilcoxon rank test (P<0.05).

### 2.7 Clinical samples

This study included 79 patients (18 females and 61 males) with primary LUSC who underwent surgical treatment at the First Affiliated Hospital of Kunming Medical University from 2009 to 2015. The age ranged from 40 to 76 years. None of the patients had undergone other treatment before surgical. All patients were free from infectious diseases. The samples were tumor tissue from LUSC patients. Since this study is a retrospective study, we conducted follow-ups with patients via telephone. All patients included in the study agreed to participate in this research. The Ethics Committee of First Affiliated Hospital of Kunming Medical University approved the study protocol (Ethics Batch No. 2024 L 183). Clinical procedures were in accordance with the Declaration of Helsinki.

### 2.8 Immunohistochemistry (IHC)

The 4μm paraffin tissue sections were toasted at 70°C for 2h; the xylene was dewaxed; and the gradient alcohol was hydrated. Citrate solution (pH 6.0) was used to repair the antigen. Endogenous peroxidase was blocked by hydrogen peroxide (0.3%) at room temperature, and non-specific antigen was blocked by sheep serum (2.5%). Sections were incubated with rabbit CSF3R、FGA、ORM1 and NTS antibody at 30°C for 80 minutes. Sections were stained with solution A in DAB chromogenic solution, hematoxylin was used to stain the nuclei, and neutral gum was used to seal the sections. A score of > 4 was considered positive. The following antibodies were used in this study: CSF3R (Cusabio, China); NTS (821026, Proteintech, China); ORM1 (16439-1-AP, ZEN-BIOSCIENCE, China); FGA (20645-1-AP, Proteintech, China).

### 2.9 Statistical analysis

Data were statistically analyzed using R3.6.0. The Shapiro-Wilk (S-W) test showed that the measures did not conform to normal distribution, so the Wilcoxon rank sum test was used for comparisons between multiple groups. Kaplan-Meier curves were used to evaluate survival differences between groups. One-way Cox analysis was used to screen prognostic factors, and multifactor Cox analysis was used to establish a regression model, in which the likelihood ratio test was used for hypothesis testing of the regression coefficients and backward selection was used for variable screening. The test level α = 0.05 (two-tailed). A chi-squared test was used to analyze the correlation between m7g immune-related gene expression in LUSC tissues and clinicopathological parameters. Non-parametric test was used to analyze the correlation between m7g-immune gene expression in LUSC tissues and patient age. OS rates were analyzed with log-rank test (Kaplan-Meier survival analysis). P < 0.05 was considered statistically significant (* P < 0.05).

## 3 Results

### 3.1 Lung squamous carcinoma disease differential gene expression results

#### 3.1.1 Differential gene expression analysis

**Figure 2:**
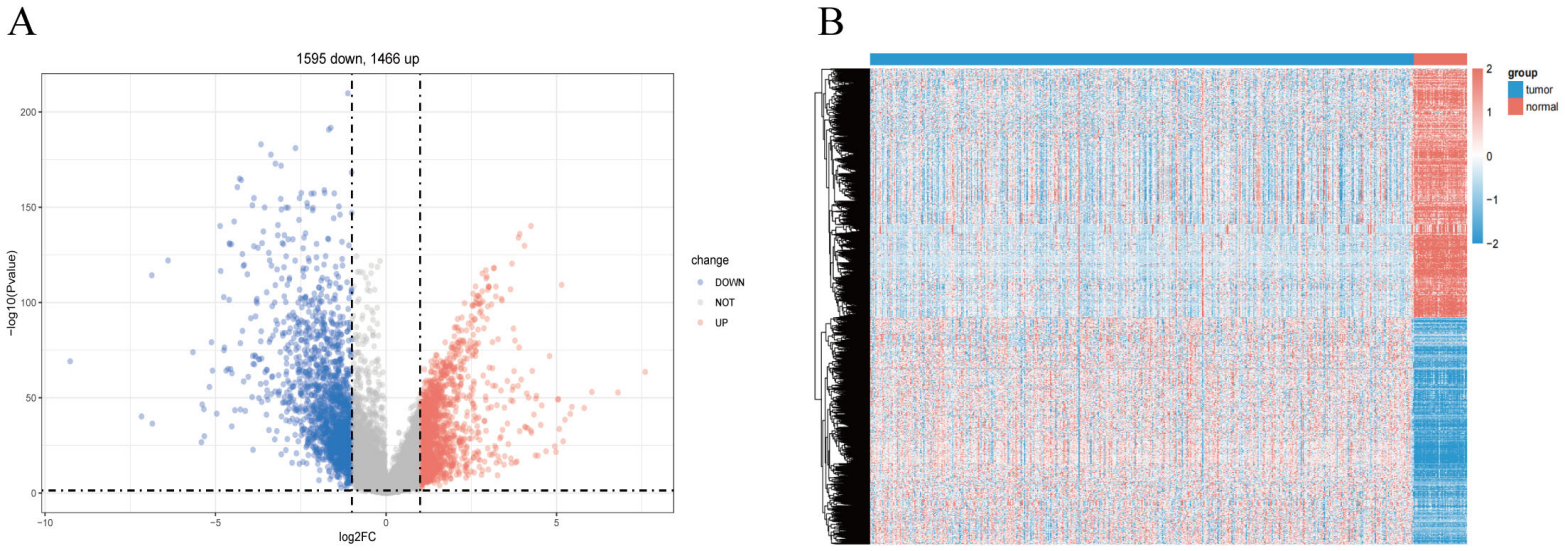
Screening for disease-differentiated genes in LUSC. **(A)** The volcano plot comparing lung squamous cell carcinoma (LUSC) samples with normal lung tissue highlights 3,061 differentially expressed genes. **(B)** Heatmap showing the differentially expressed genes between lung squamous cell carcinoma (LUSC) and normal lung tissue.

To identify LUSC-related genes, we used the limma package to analyze transcriptome data from 49 control and 501 LUSC cases. Differential criteria included P <0.05 and |Log2FC|>0.5. From a statistical standpoint, a total of 3,061 exhibited significant differential express ion in LUSC compared to the normal tissue, which 1,466 genes being up-regulated and 1,595 genes being down-regulated and The volcano plot (**Figure 2A**) visualized these differential expressions. Additionally, individual sample gene expression profiles (**Figure 2B**) revealed a predominance of up-regulated genes in tumor tissues and down-regulated genes in normal tissues.

#### 3.1.2 Weighted Gene Co-expression Network Analysis (WGCNA)

Our practical WGCNA analysis to find disease-related genes (**Supplementary Figure S1A**), as shown in (**Supplementary Figure S1B**), we chose the optimal threshold as 5. By constructing the co-expression network from (**Supplementary Figure S1C**), we can see that a total of 8 modules were grouped together except for the gray modules, and we found that MEblue, MEturquoise showed the highest correlation with clinical traits at a significant level (p<0.05). **Supplementary Figure S1D** shows that MEblue and ME-turquoise showed the highest correlation with clinical traits at a significant level (p<0.05). This module will be selected for subsequent analysis, the MEblue module contains 5062 modular genes and the MEturquoise module contains 6705 modular genes.

### 3.2 Differential module M7G immune gene screening and target gene function enrichment

**Figure 3:**
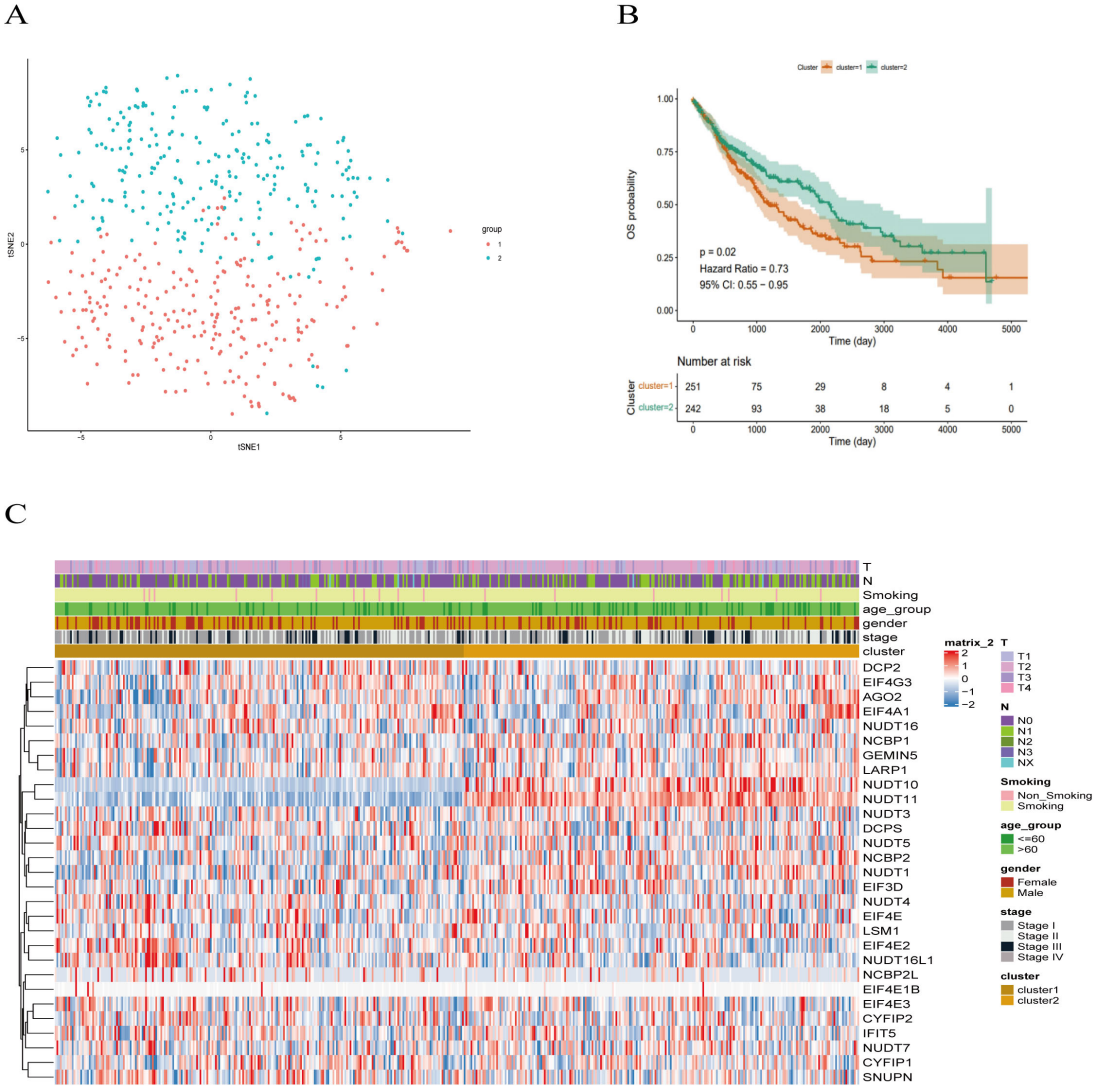
Clinical trait analysis of m7G-associated LUSC subtypes. **(A)** Results of two LUSC m7G clusters: Cluster 1 and Cluster 2 are clearly separated, and the results are reliable. **(B)** Kaplan-Meier overall survival curve for two LUSC m7G clusters: overall survival differs between Cluster 1 and Cluster 2 (P < 0.05), with Cluster 2 showing better survival than Cluster 1. **(C)** Clinical features of the two LUSC m7G clusters: N stage, gender, and overall stage differ among different subtypes.

Based on the 29 m7G methylation-regulated genes, ConsensusClusterPlus R package was used for consistent clustering analysis. The cumulative distribution function (CDF) curve decreased most slowly when K = 2 (**Supplementary Figure S2A**), so we determined that the LUSC samples were clustered into two subclasses, and the heatmap of the clustering matrix at K = 2 was clearly divided into two blocks, cluster 1 contained 251 patients cluster 1 contains 251 patients, and cluster 2 contains 242 patients. The clustering results were evaluated using t-SNE dimensionality reduction analysis (**Supplementary Figure S2B**), which showed that cluster 1 and cluster 2 were clearly separated (**Figure 3A**), predicting reliable consistent clustering results.

Next, we applied Kaplan-Meier analysis to analyze the prognostic value of this clustering. The results revealed a significant difference in overall survival between the Cluster 1 and Cluster 2 subclasses (P < 0.05), with Cluster 2 exhibiting better survival outcomes than Cluster 1 (**Figure 3B**). Subsequently, we compared the clinical features and gene expression profiles of the different subclasses. As shown in (**Figure 3C**), there were significant differences in N-stage, gender, and stage between the subtypes, whereas changes in other clinicopathological features were not statistically significant between the clusters. We found that m7G methylation-regulated genes were potentially correlated with lymphatic metastasis, gender, and tumor stage in LUSC.

**Figure 4:**
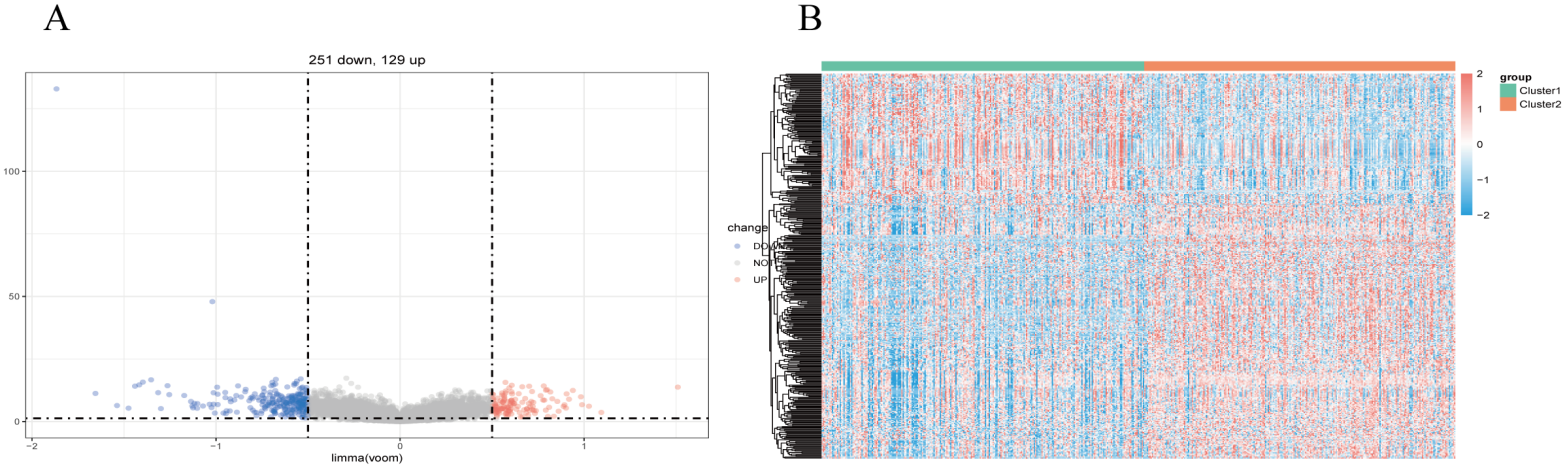
Identification of m7G-associated LUSC genes. **(A)** A total of 380 differentially expressed genes were found in two LUSC m7G clusters, including 129 upregulated genes and 251 downregulated genes. **(B)** Expression of m7G-associated genes in two LUSC m7G clusters.

We utilized the limma package for differential analysis of M7G isoform-related genes, applying differential screening conditions of P <0.05 and |Log2FC|>0.5. In total, 380 genes found to be statistically significantly differentially expressed between cluster 1 and cluster 2, with 129 genes up-regulated and 251 genes were down-regulated (**Figure 4A**). These differences are illustrated in the heatmap of M7G-related genes (**Figure 4B**).

**Figure 5:**
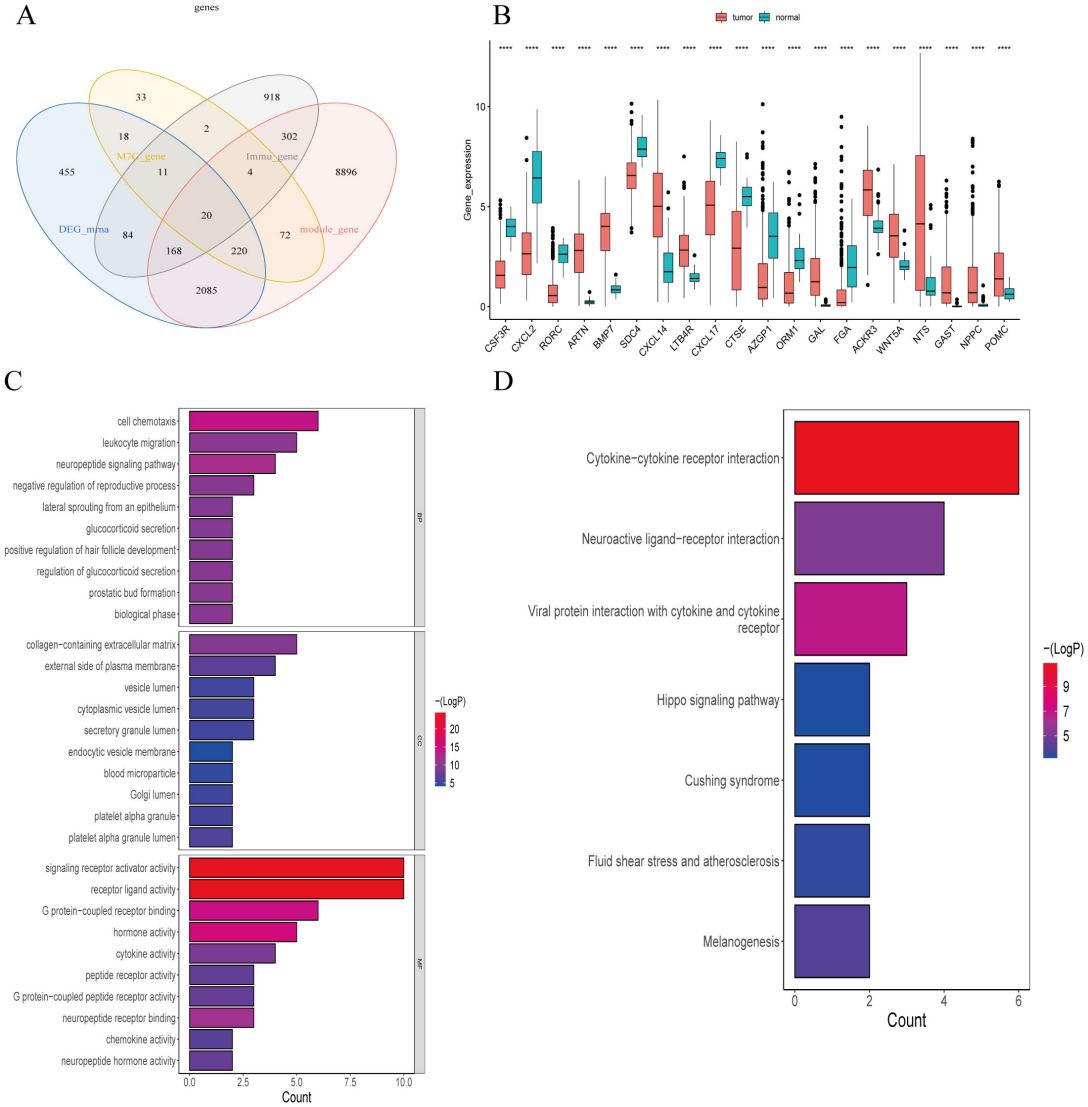
Screening and enrichment analysis of m7G-Related Immune Genes in Lung Squamous Cell Carcinoma (LUSC). **(A)** Venn diagram of differential gene screening from four LUSC m7G-associated immune clusters: 20 overlapping genes were identified. **(B)** Expression of 20 m7G-related immune genes in tumor and normal groups. **(C)** The 20 m7G-related immune genes in LUSC were enriched in 284 GO terms, with the top 23 GO pathways shown. **(D)** The 20 m7G-related immune genes in LUSC were enriched in seven KEGG pathways. ****P<0.0001.

We used a Ven diagram (**Figure 5A**) to illustrate the intersection of LUSC and normal differential genes, modular genes, and M7G-related genes with the 2013 immunization genes from the Immport database. The results identified 20 intersecting genes, which were subsequently analyzed. The expression of the 20 genes in the disease and normal groups is shown in **Figure 5B**, in which ARTN, BMP7, CXCL14, LTB4R, GAL, ACKR3, WNT5A, NTS, GAST, NPPC, and POMC were significantly up-regulated in tumor tissues as compared to normal tissues.

Our GSVA enrichment analysis of 20 differential modular M7G Immune-related genes based on KEGG gene set, A total of 284 GO entries were generated. **Figure 5C** highlights the top23 GO pathways, including biological process (BP), cellular component (CC), and molecular function (MF) categories. Notable entries include receptor-ligand activity, signaling receptor activation activity, cell chemotaxis, G-protein-coupled receptor binding, leukocyte migration, collagen-containing extracellular matrix, hormone activity, neuropeptide signaling pathway, leukocyte chemotaxis, connective tissue development, second-messenger-mediated signaling, cell adhesion positive regulation, cytokine-mediated signaling pathway, positive regulation of MAPK cascade, cyclic-nucleotide-mediated signaling, etc.

KEGG enrichment results demonstrated enrichment in seven KEGG pathways (**Figure 5D**), including cytokine-cytokine receptor interactions, viral proteins interacting with cytokines and cytokine receptors, interactions in the stimulation of neural tissue, bactericidal effects, fluid shear stress, and atherosclerosis pathways.

### 3.3 Construction and validation of immune-related genes risk models

**Figure 6:**
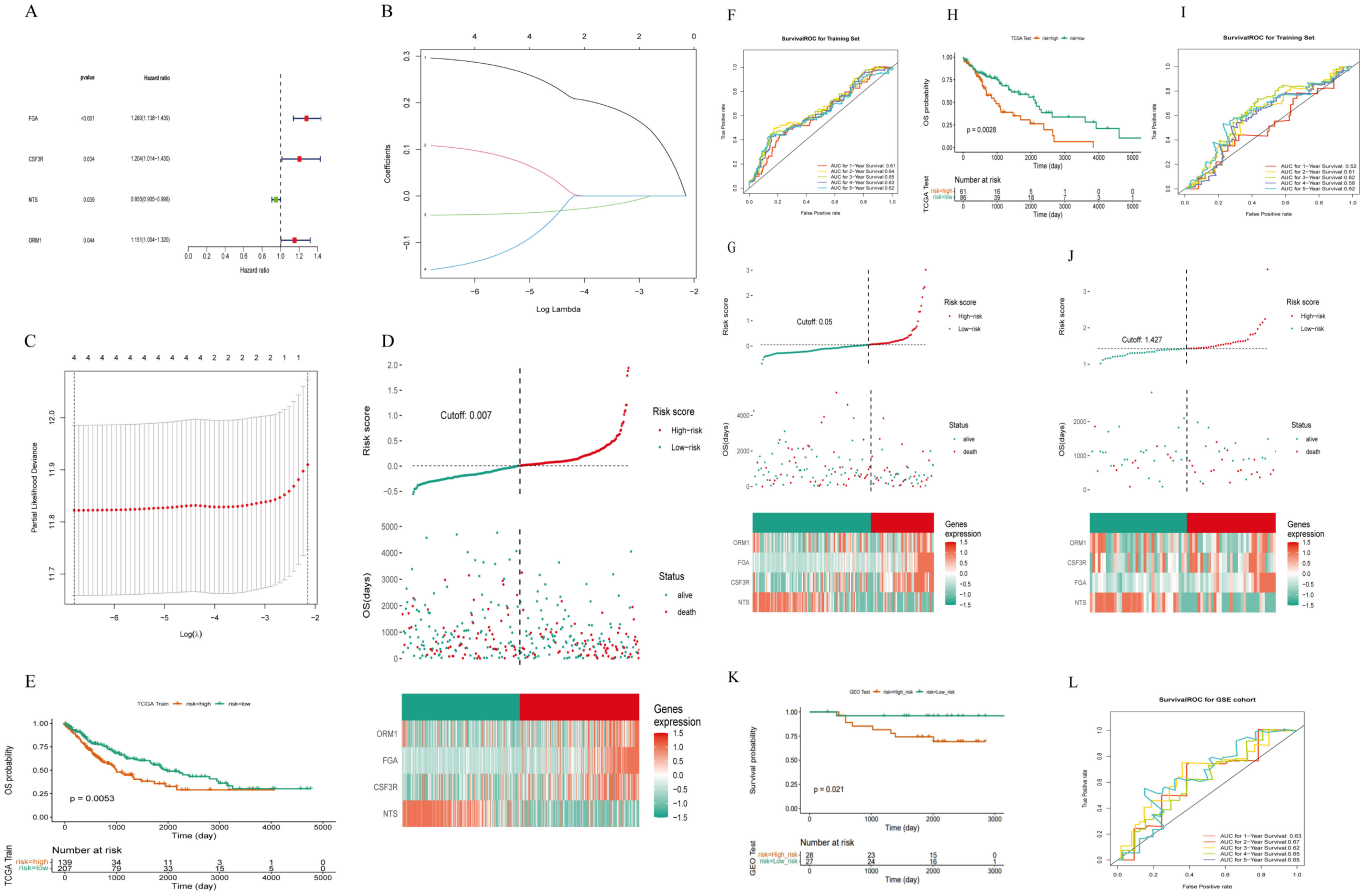
Construction of a risk model of m7g immune-related genes in LUSC. **(A)** Four prognosis-related genes identified by univariate Cox regression analysis: 20 genes were initially screened out and four genes were associated with survival (P< 0.05). **(B)** LASSO coefficient distribution of four m7G immune-related genes in LUSC. **(C)** The tuning parameter (λ) in the LASSO model was selected using the minimum criterion. **(D)** LUSC patients in the TCGA-LUSC training set were divided into high-risk and low-risk groups based on risk scores. **(E)** Survival analysis of the TCGA-LUSC training set: the high-risk group had worse survival compared to the low-risk group. **(F)** ROC curve for the TCGA-LUSC training set: the AUC values for 1-, 3-, and 5-year survival were all greater than 0.60. **(G)** Risk curves, scatter plots, high- and low-risk group models, and gene expression heatmap for the TCGA-LUSC internal validation set. **(H)** Survival analysis for the TCGA-LUSC internal validation set: the high-risk group had lower survival rates. **(I)** ROC curve for the TCGA-LUSC internal validation set. **(J)** The GEO External dataset GSE50081 risk curves, scatter plots, set Heatmap and modeled gene expression for high- and low-risk subgroups. **(K)** Survival analysis of the GEO external validation dataset GSE50081: the high-risk group had worse survival compared to the low-risk group. **(L)** ROC curve for the GEO external validation dataset GSE50081: the AUC values for 1-, 3-, and 5-year survival were all greater than 0.60.

We employed the 20 genes previously identified and utilized the expression data (FPKM) to divide the TCGA-LUSC comprising 493 samples after excluding those with incomplete survival information, into training and validation sets in a 7:3 ratio. The training set consisted of 346 cases, while the validation set comprised 147 cases. Univariate analysis results in the training set are depicted in **Figure 6A**, revealing that four out of 20 genes exhibited a P<0.05. Specifically, FGA, CSF3R, and ORM1 were identified as risk genes, whereas NTS was identified as a protective gene.

Subsequently, the four selected genes underwent further screening using the LASSO regression analysis in the training set. **Figures 6B, C** illustrates that FGA, CSF3R, NTS, and ORM1 were retained in the model due to their lowest cross-validation error. Patients were then assigned risk scores based on the expression levels of these four model genes and their corresponding coefficients obtained from lasso regression. Using the median risk score as a threshold (**Figure 6D**), patients were stratified into high-risk and low-risk groups. As shown in (**Figure 6E**): LUSC patients in the high-risk group of the training set had worse survival.

The risk score was calculated by the formula:


$$\begin{array}{c} \text{RiskScore}\;=\;\text{FGA}\times 0.29604+\text{CSF}3\text{R}\times 0.10820\;+\text{NTS}\\ \times \left(-0.04118\right)+\;\text{ORM}1\times 0.15818 \end{array}$$

Risk curves based on risk scores, and **Figure 6F** demonstrated significantly higher survival rates in the low-risk group compared to the high-risk group. The area under the curve (AUC) for predicting 1-, 3-, and 5-year survival rates were all greater than 0.60, respectively with 0.61, 0.65, and 0.62.

Upon constructing the risk model, internal validation was performed using the validation set from TCGA. Risk curves, scatter plots (**Figure 6G**), heat maps depicting gene expression in high and low-risk groups, and Kaplan-Meier survival curves (**Figure 6H**) confirmed distinct survival outcomes between the two risk groups. Similarly, ROC curves for the internal validation set (**Figure 6I**) indicated AUCs > 0.60 for predicting 1- and 3-year survival rates.

Furthermore, external validation of the risk model was conducted on 55 LUSC with intact overall survival (OS) information from the GEO external GSE50081 dataset. Consistent results were obtained, as depicted in **Figures 6J–L**, affirming the robust prognostic capability of the newly developed risk model for LUSC patients.

**Figure 7:**
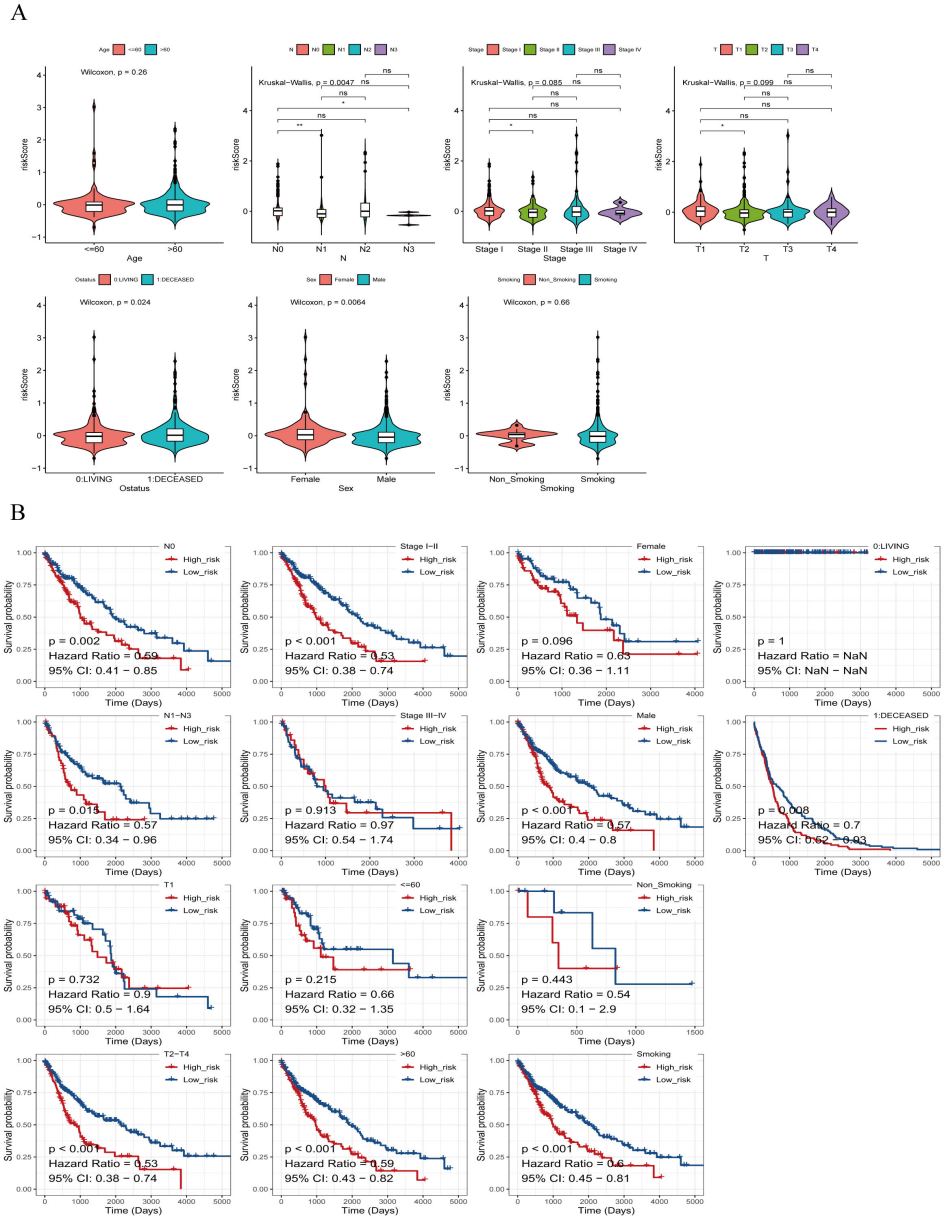
Risk scores of different risk groups. **(A)** Based on age, the patients were divided into: >60 and ≤60 subgroups. Risk scores were significantly different in Ostatus, N stage, and gender groups, but not in other groups. **(B)** Survival analysis based on clinical factors was performed on the risk groups. Except for T1, stage IIIs-IV, ≤60, females, and non-smokers, other clinical features were statistically significant. *P<0.05, **P<0.01, ns indicates P > 0.05 (representing no statistically significant difference).

Additionally, we explored associations between risk scores and clinicopathologic characteristics. Patients were categorized by age into (>60 vs. ≤60), and differences in risk scores across clinical subgroups were analyzed. **Figure 7A** shows significantly differences in risk scores among subgroups based on Ostatus (Overall Survival Status), N-stage, and gender. We also investigated stratified survival analyses of risk models of m7G immune-associated genes in combination with various clinical features (**Figure 7B**). The analysis revealed significant differences across most clinical traits, except for clinical stage T1, Stage III-IV, ≤60, Female, and Non-Smoking, where no significant differences were observed between the high and low risk groups. Notably, ≤60-year-old patients had significantly lower risk scores compared to patients aged > 60 years, suggesting a better prognosis in the younger group.

### 3.4 Comparison of the construction and prognostic characteristics of nomograms

**Figure 8:**
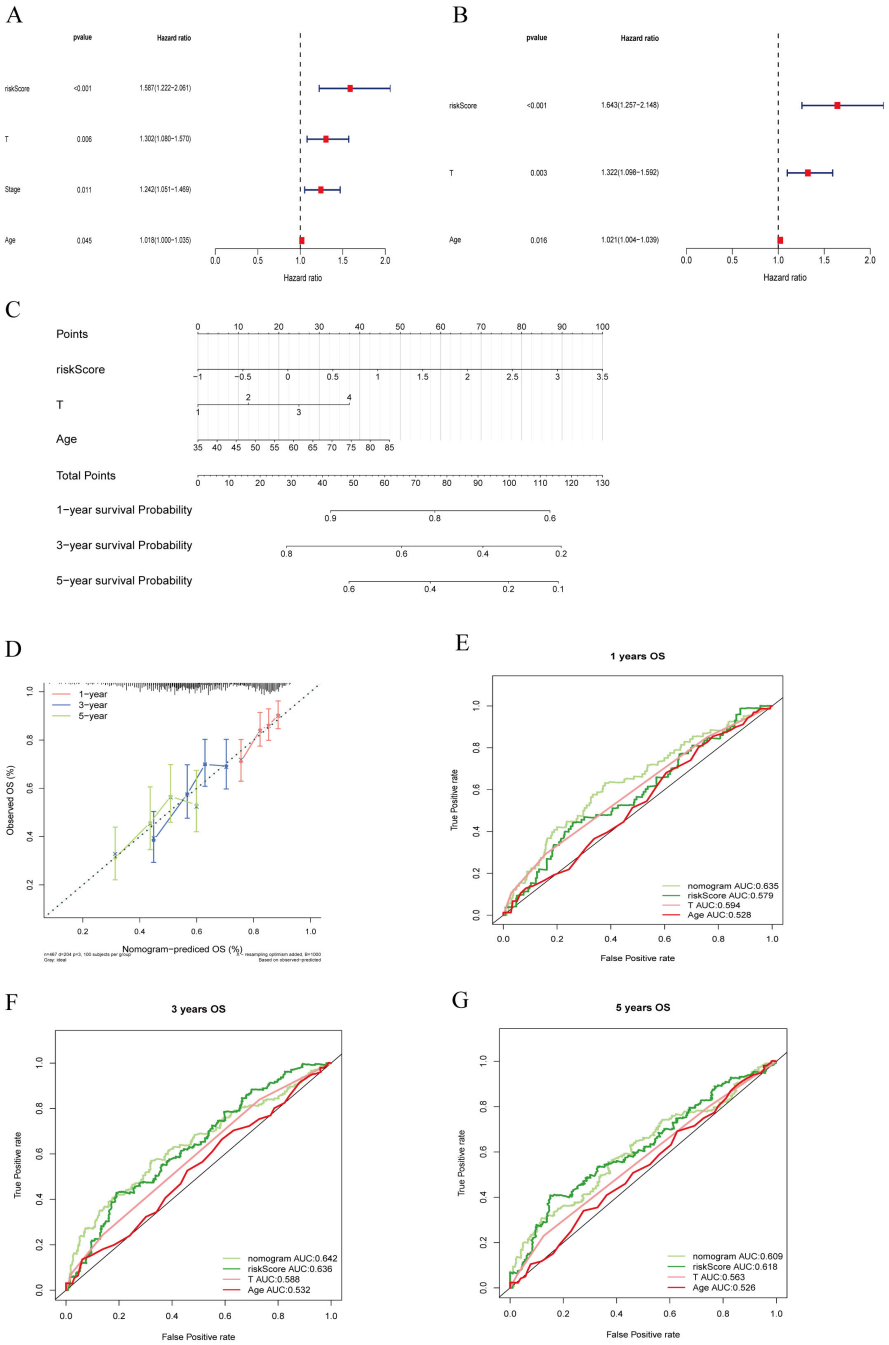
Clinical value of risk characteristics in TCGA-LUSC. **(A)** Univariate Cox regression analysis shows that T stage, overall Stage, age, and risk score factors are independent prognostic factors. **(B)** Multivariate Cox regression analysis of risk scores and clinical factors. T stage, age, and risk score are independent prognostic factors. **(C)** A nomogram combining age, T stage, and risk score predicts 1-, 3-, and 5-year survival probability. **(D)** Calibration curves test the agreement between actual and predicted results at 1, 3, and 5 years. **(E–G)** ROC curve analysis of independent prognostic models at 1, 3, and 5 years.

To develop a practical clinical assessment tool to improve the accuracy of predicting OS in individuals with LUSC, we constructed a nomogram containing age, gender, T-stage, tumor stage, and risk score, and conducted a univariate Cox proportional hazards analysis to identify independent prognostic factors (**Figure 8A**).

Multivariate Cox regression analysis (**Figure 8B**) identified T stage, age, and riskScore as independent prognostic factors (P < 0.05).

Subsequently, we constructed an independent prognostic model using the clinical factors riskScore, T stage, Age. The nomogram (**Figure 8C**) visualizes this predictive model and estimates the 1-, 3-, and 5-year probabilities for patients with LUSC. The calibration plot (**Figure 8D**) confirms that the predicted 1-, 3-, and 5-year survival rates are consistent with the actual observed results. These findings suggest that T stage, clinical stage, age, and RiskScore are important factors in clinical evaluation of LUSC patient prognosis. To ensure comparability between models, we calculated the area under the ROC curve for the independent prognostic predictor (risk score, T-stage, and age) in the entire TCGA cohort. The results showed that the inclusion of these three independent prognostic factors constituted the model with the highest AUC value, which was superior to the model based on a single factor. Specifically, the riskScore+T-stage+Age model achieved the highest AUC values for predicting 1- and 3-years survival rates (**Figures 8E–G**), indicating that this combination provided the most accurate prognostic model.

Therefore, our findings underscore the superior effective of the nomogram incorporating risk score, T-stage, and age in estimating the prognosis for patients with LUSC.

### 3.5 Expression of M7G immune-related genes in lung squamous carcinoma specimens and their prognostic implications

**Figure 9:**
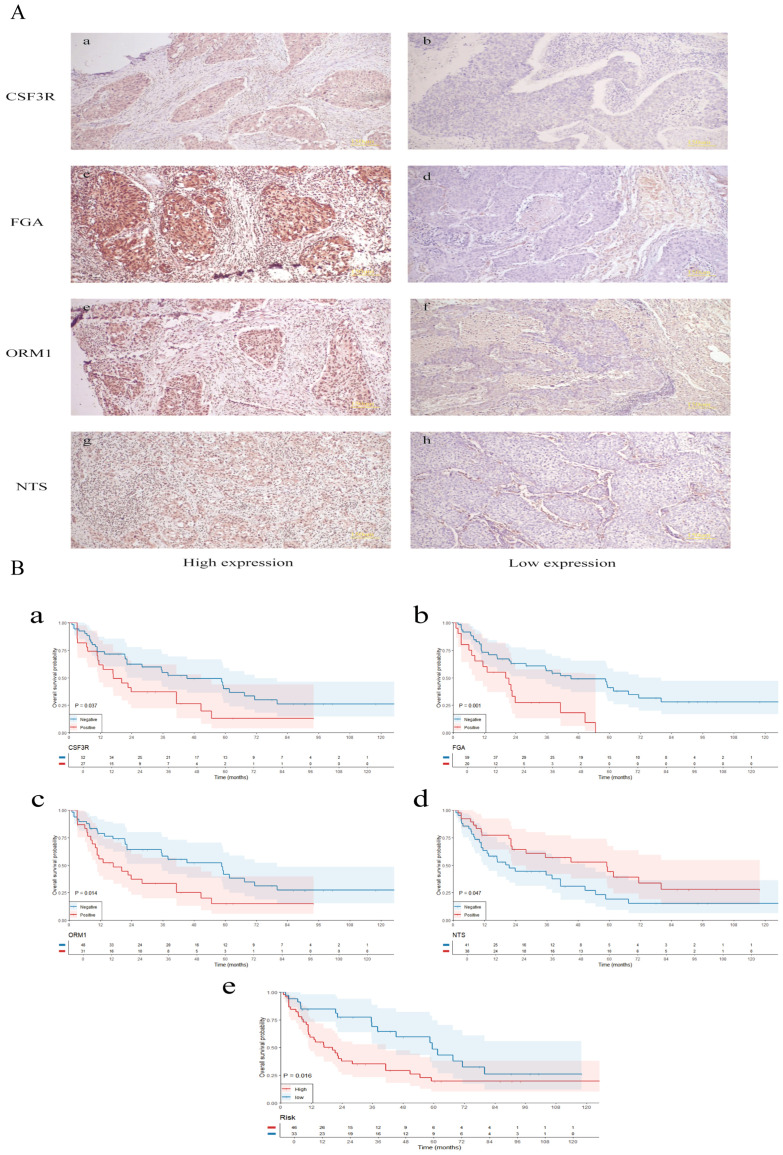
Validation of clinical samples. **(A)** Immunohistochemistry was employed to detect the expression levels of FGA, CSF3R, ORM1, and NTS. **(B)** Survival analysis of m7G immune-related gene expression, risk stratification, and their correlation with the prognosis of lung squamous cell carcinoma patients. The small letters indicates the scale bar that represents 150 μm.

To investigate the expression of M7G immune-related genes in clinical lung squamous cell carcinoma (LUSC) specimens and their correlation with patient prognosis, we collected 79 postoperative paraffin-embedded samples from LUSC patients who underwent surgery at the First Affiliated Hospital of Kunming Medical University. Follow-ups with patients were conducted via telephone. Immunohistochemistry (**Figure 9A**) was employed to detect the expression levels of FGA, CSF3R, ORM1, and NTS.

Our results showed no significant correlation between the expression of these genes and the patients’ gender, age, smoking status, N stage, T stage, or overall clinical stage. However, a positive correlation was observed between the expression of FGA and CSF3R (P=0.002) and ORM1 (P<0.001), while FGA expression was negatively correlated with NTS (P=0.002, **Table 1**). CSF3R expression was positively correlated with FGA(P=0.002) and ORM1 (P=<0.001, **Table 2**), had no significant correlation with NTS (P=0.098, **Table 3**). Furthermore, ORM1 expression was negatively correlated with NTS (P=0.013, **Table 4**).

**Table 1:** Correlation between the clinicopathological characteristics and expression of FGA in LUSC.

Characteristics	Negative (n=59)	Positive (n=20)	Total (n=79)	P value
Age, M(IQR)	67.0 (61.5,71.0)	66.5 (57.0,70.0)	67.0 (60.5,70.5)	0.848
Gender, n(%)				0.373
Female	12 (20.3%)	6 (30.0%)	18 (22.8%)	
Male	47 (79.7%)	14 (70.0%)	61 (77.2%)	
Smoking, n(%)				0.767
Quit smoking ≤15 years	44 (74.6%)	16 (80.0%)	60 (75.9%)	
Quit smoking > 15 years	15 (25.4%)	4 (20.0%)	19 (24.1%)	
N, n(%)				0.232
0	40 (67.8%)	17 (85.0%)	57 (72.2%)	
1-3	19 (32.2%)	3 (15.0%)	22 (27.8%)	
T, n(%)				0.434
1	15 (25.4%)	5 (25.0%)	20 (25.3%)	
2	34 (57.6%)	14 (70.0%)	48 (60.8%)	
3 + 4	10 (16.9%)	1 (5.0%)	11 (13.9%)	
Stage, n(%)				0.076
I	32 (54.2%)	16 (80.0%)	48 (60.8%)	
II+III	27 (45.8%)	4 (20.0%)	31 (39.2%)	
CSF3R, n(%)				0.002*
Negative	45 (76.3%)	7 (35.0%)	52 (65.8%)	
Positive	14 (23.7%)	13 (65.0%)	27 (34.2%)	
NTS, n(%)				0.002*
Negative	24 (40.7%)	17 (85.0%)	41 (51.9%)	
Positive	35 (59.3%)	3 (15.0%)	38 (48.1%)	
ORM1, n(%)				<0.001***
Negative	43 (72.9%)	5 (25.0%)	48 (60.8%)	
Positive	16 (27.1%)	15 (75.0%)	31 (39.2%)	

*P<0.05, ***P<0.001.

**Table 2:** Correlation between the clinicopathological characteristics and expression of CSF3R in LUSC.

Characteristics	Negative (n=52)	Positive (n=27)	Total (n=79)	P value
Age, M(IQR)	67.0 (59.5,70.5)	67.0 (61.5,70.5)	67.0 (60.5,70.5)	0.967
Gender, n(%)				0.058
Female	8 (15.4%)	10 (37.0%)	18 (22.8%)	
Male	44 (84.6%)	17 (63.0%)	61 (77.2%)	
Smoking				0.576
Quit smoking ≤15 years	41 (78.8%)	19 (70.4%)	60 (75.9%)	
Quit smoking > 15 years	11 (21.2%)	8 (29.6%)	19 (24.1%)	
N, n(%)				0.992
0	37 (71.2%)	20 (74.1%)	57 (72.2%)	
1-3	15 (28.8%)	7 (25.9%)	22 (27.8%)	
T, n(%)				0.837
1	12 (23.1%)	8 (29.6%)	20 (25.3)	
2	32 (61.5%)	16 (59.3%)	48 (60.8)	
3 + 4	8 (15.4%)	3 (11.1%)	11 (13.9)	
Stage, n(%)				0.595
I	30 (57.7%)	18 (66.7%)	48 (60.8%)	
II+III	22 (42.3%)	9 (33.3%)	31 (39.2%)	
NTS, n(%)				0.098
Negative	23 (44.2%)	18 (66.7%)	41 (51.9%)	
Positive	29 (55.8%)	9 (33.3%)	38 (48.1%)	
ORM1, n(%)				<0.001***
Negative	40 (76.9%)	8 (29.6%)	48 (60.8%)	
Positive	12 (23.1%)	19 (70.4%)	31 (39.2%)	
FGA, n(%)				0.002*
Negative	45 (86.5%)	14 (51.9%)	59 (74.7%)	
Positive	7 (13.5%)	13 (48.1%)	20 (25.3%)	

*P<0.05, ***P<0.001.

**Table 3:** Correlation between the clinicopathological characteristics and expression of ORM1 in LUSC.

Characteristics	Negative (n=48)	Positive (n=31)	Total (n=79)	P value
Age, M(IQR)	67.5 (64.5,72.0)	65.0 (58.5,68.0)	67.0 (60.5,70.5)	0.114
Gender, n()				>0.999
Female	11 (22.9%)	7 (22.6%)	18 (22.8%)	
Male	37 (77.1%)	24 (77.4%)	61 (77.2%)	
Smoking				0.606
Quit smoking ≤15 years	35 (72.9%)	25 (80.6%)	60 (75.9%)	
Quit smoking > 15 years	13 (27.1%)	6 (19.4%)	19 (24.1%)	
N, n(%)				>0.999
0	35 (72.9%)	22 (71.0%)	57 (72.2%)	
1-3	13 (27.1%)	9 (29.0%)	22 (27.8%)	
T, n(%)				0.801
1	11 (22.9%)	9 (29.0%)	20 (25.3%)	
2	30 (62.5%)	18 (58.1%)	48 (60.8%)	
3 + 4	7 (14.6%)	4 (12.9%)	11 (13.9%)	
Stage, n(%)				0.754
I	28 (58.3%)	20 (64.5%)	48 (60.8%)	
II+III	20 (41.7%)	11 (35.5%)	31 (39.2%)	
CSF3R, n(%)				<0.001***
Negative	40 (83.3%)	12 (38.7%)	52 (65.8%)	
Positive	8 (16.7%)	19 (61.3%)	27 (34.2%)	
NTS, n(%)				0.013*
Negative	19 (39.6%)	22 (71.0%)	41 (51.9%)	
Positive	29 (60.4%)	9 (29.0%)	38 (48.1%)	
FGA, n(%)				<0.001***
Negative	43 (89.6%)	16 (51.6%)	59 (74.7%)	
Positive	5 (10.4%)	15 (48.4%)	20 (25.3%)	

*P<0.05, ***P<0.001.

**Table 4:** Correlation between the clinicopathological characteristics and expression of NTS in LUSC.

Characteristics	Negative (n=41)	Positive (n=38)	Total (n=79)	P value
Age, M(IQR)	68.0 (62.0,72.0)	66.0 (56.0,68.0)	67.0 (60.5,70.5)	0.058
Gender, n(%)				>0.999
Female	9 (22.0%)	9 (23.7%)	18 (22.8%)	
Male	32 (78.0%)	29 (76.3%)	61 (77.2%)	
Smoking, n(%)				>0.999
Quit smoking ≤15 years	31 (75.6%)	29 (76.3%)	60 (75.9%)	
Quit smoking > 15 years	10 (24.4%)	9 (23.7%)	19 (24.1%)	
N, n(%)				0.335
0	32 (78.0%)	25 (65.8%)	57 (72.2%)	
1-3	9 (22.0%)	13 (34.2%)	22 (27.8%)	
T, n(%)				0.878
1	11 (26.8%)	9 (23.7%)	20 (25.3%)	
2	25 (61.0%)	23 (60.5%)	48 (60.8%)	
3 + 4	5 (12.2%)	6 (15.8%)	11 (13.9%)	
Stage, n(%)				0.233
I	28 (68.3%)	20 (52.6%)	48 (60.8%)	
II+III	13 (31.7%)	18 (47.4%)	31 (39.2%)	
CSF3R, n(%)				0.098
Negative	23 (56.1%)	29 (76.3%)	52 (65.8%)	
Positive	18 (43.9%)	9 (23.7%)	27 (34.2%)	
ORM1, n(%)				0.013*
Negative	19 (46.3%)	29 (76.3%)	48 (60.8%)	
Positive	22 (53.7%)	9 (23.7%)	31 (39.2%)	
FGA, n(%)				0.002*
Negative	24 (58.5%)	35 (92.1%)	59 (74.7%)	
Positive	17 (41.5%)	3 (7.9%)	20 (25.3%)	

*P<0.05.

Survival analysis (**Figure 9B**) revealed that high expression levels of CSF3R, ORM1, and FGA were associated with poorer prognosis in LUSC patients, whereas high expression of NTS was associated with better prognosis. Based on the expression levels of FGA, CSF3R, NTS, and ORM1, patients were stratified into high-risk and low-risk groups. The high-risk group was significantly associated with poorer prognosis.

### 3.6 Research on immune microenvironment and anti-cancer therapy

**Figure 10:**
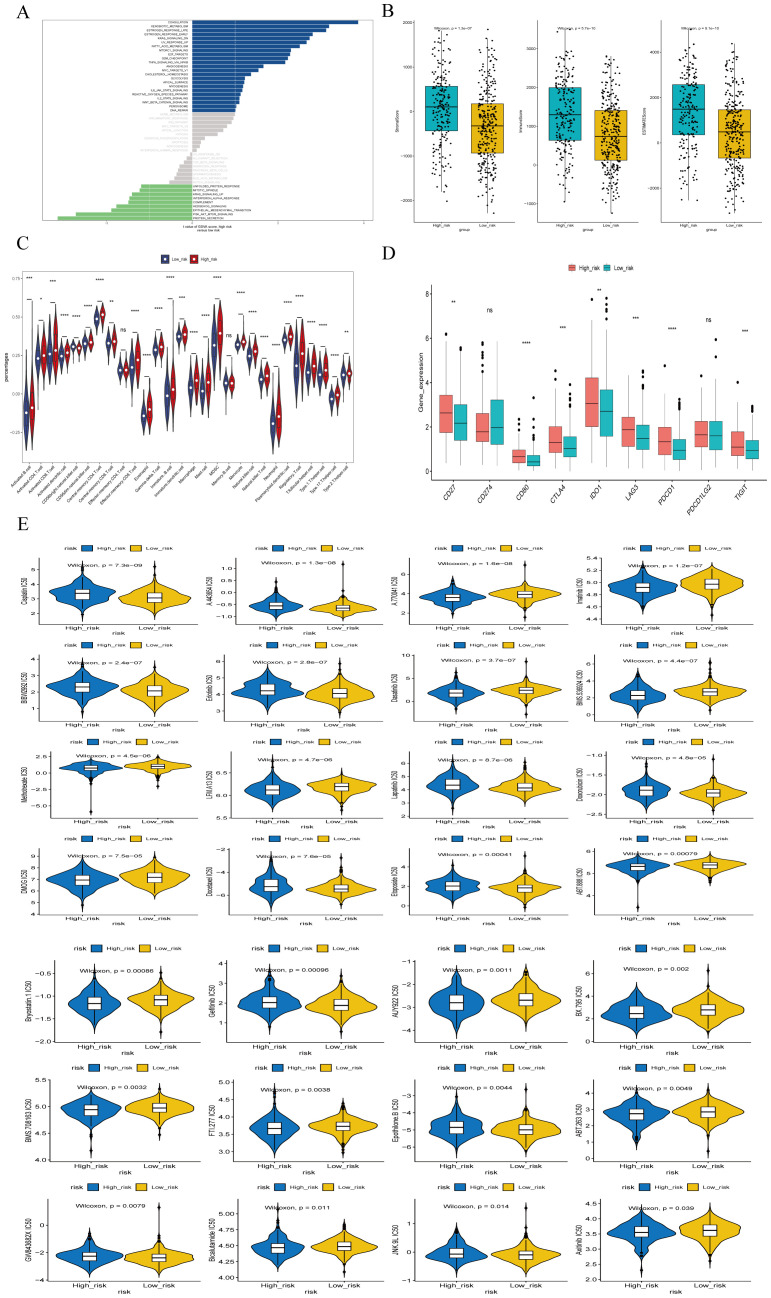
Enrichment analysis of different risk subgroups. **(A)** GSVA pathway enrichment analysis of different risk subgroups. **(B)** ESTIMATE scores of different risk subgroups. **(C)**Violin plot of immune cell scores in high- and low-risk groups based on the ssGSEA algorithm. **(D)** Differences in immune checkpoints between high- and low-risk groups. **(E)** Drug sensitivity analysis for high- and low-risk groups. *P<0.05, **P<0.01, ***P<0.001, ****P<0.0001, ns indicates P > 0.05 (representing no statistically significant difference).

We performed GSVA analysis on samples from the high-risk and low-risk groups. as shown in **Figure 10A**, the high-risk group exhibited activation in 23 pathways, including coagulation, E2F targets, G2M checkpoint, IL6/JAK/STAT3 signaling, DNA repair, myogenesis, estrogen response, peroxisome, angiogenesis, TGFβ signaling, and MYC target V1. pathways, including the low-risk group showed activated in 9 pathways, including protein secretion, PI3K/AKT/mTOR signaling, interferon alpha response, mitotic spindle, and unfolded protein response.

Tumor microenvironment (TME) cells are a crucial component of tumor tissues, and an increasing body of evidence highlights their Clinical significance in predicting prognosis and treatment outcome. We inferred the proportion of stromal and immune cells in tumor samples based on gene expression, assigning each sample 3 scores: stromal score, immune score and ESTIMATE composite score. Additionally, we calculated the immune cell in the samples using the ssGSEA algorithm. The results indicated that the stromal score, immune score and ESTIMATE composite score were significantly lower in the high-risk group compared to the low-risk group (**Figure 10B**). The ssGSEA algorithm results (**Figure 10C**) showed that the scores of 26 immune cells, including activated B cells, activated CD4^+^ T cells, activated CD8^+^ T cells, CD56 natural killer cells, and mast cells, were significantly lower in the high risk groups. Patients in the low-risk group had higher immune scores than those in the high-risk group, reflecting differences immune function between the two groups.

We also performed a differential analysis of the expression of 9 Immune Checkpoints between the high- and low-risk groups, with significant differences observed in loci such as CD27, CD80, CTLA4, PDCD1 (**Figure 10D**). Furthermore, we investigated common drug sensitivity, demonstrating significant differences in response to chemotherapeutic drugs agents, such as Lapatiniband Gefitinib (P < 0.05), as shown in **Figure 10E**.

## 4 Discussion

Lung cancer is one of the leading causes of cancer-related deaths worldwide (24). It is broadly categorized into two main subtypes: small cell lung cancer (SCLC) and non-small cell lung cancer (NSCLC), with NSCLC constituting approximately 85% of cases. Among NSCLC subtypes, lung adenocarcinoma (LUAD) and lung squamous cell carcinoma (LUSC) are the most common, with LUSC accounting for 40% of all cases (25). Despite advancements in surgery, radiotherapy, targeted therapy, and immunotherapy, the 5-year survival rate of lung cancer patients remains discouragingly low (3). Compared to Lung adenocarcinoma (LUAD), LUSC presents a poor clinical prognosis and lacks targeted drugs. The role of m7G in tumor has garnered increasing attention, yet there are currently no report on the involvement of m7G immune-related genes in lung squamous cell carcinoma (LUSC) and their impact on the prognosis and immunotherapy. Therefore, our aim is to screen m7G immune-related genes and construct a risk model to assess the prognosis of patients with LUSC.

We intersected LUSC and normal lung tissue differential genes, modular genes and M7G-related genes with 2013 immune genes in the immport database, and the results showed that a total of 20genes were extracted. Among them, ARTN, BMP7, CXCL14, LTB4R, GAL, ACKR3, WNT5A, NTS, GAST, NPPC, and POMC were highly expressed in tumor tissues.

Artemin (ARTN) is a member of glial cell line-derived neurotrophic factor (GDNF) family of ligands, ARTN triggers oncogenicity and metastasis by the activation of the AKT signaling pathway (26). Similar to other malignancies, the progression of LUSC is modulated by microenvironmental cues, including hypoxia (27).Hypoxia, one of the hallmarks of cancer, is caused by an insufficient oxygen supply, mostly due to a chaotic, deficient tumor microcirculation (28). It has been demonstrated that BMP7 has metastasis role in regulating lung cancer cell motility and invasion without influencing cell growth, proliferation, or apoptosis (29), Overexpression of BMP7 also promotes immunotherapy resistance (30).

Recently, CXCL14 has emerged as a potential diagnostic and prognostic biomarker for lung cancer patients (31).Whereas LTB4R is associated with immune cell infiltration (32), Galectins(GAL) play an active role in many types of cancer by regulating cell growth, conferring cell death resistance, or inducing local and systemic immunosuppression, allowing tumor cells to escape the host immune response (33), and it is now well established that ACKR3 plays a role in breast, lung, and brain cancers (34), ACKR3 is overexpressed in numerous cancer types and has been involved in the modulation of tumor cell proliferation and migration, tumor angiogenesis, or resistance to drugs, thus contributing to cancer progression and metastasis occurrence. A recent study showed that elevated expression of Wnt5a was associated with poor prognosis in non-small cell lung cancer (NSCLC) patients (35); Upregulation of the neuropeptide neurotrophin (NTS) is associated with poor prognosis in lung adenocarcinoma(LUAD) (36),There are relatively few studies on the prognosis of NTS and LUSC, and the correlation is not yet clear, our study shows that high NTS expression in LUSC is associated with better prognosis. and Dysregulation of GAST has also been associated with the development of various types of cancers (37),Additionally, POMC expression may be associated with tumor malignancy (38).

Subsequently, we utilized the expression data of these genes to divide the 293 samples in the TCGA- LUSC dataset into training and validation sets in a 7:3 ratio, with 206 cases in the training set and 87 cases in the validation set. Model genes were screened in the training set using univariate Cox regression followed by LASSO algorithm, identifying four feature genes at the minimum cross-validation error: FGA, CSF3R, and ORM1 as risk genes, and NTS as a protective gene. Consequently, we employed these four genes (FGA, CSF3R, ORM1 and NTS) to construct a novel prognostic model.

Comprehensive studies have shown that serum levels of FGA are upregulated in a variety of malignant tumors, including endometrial, hepatocellular, gastric, and colorectal tumors, which is consistent with our findings (39–42). CSF3R significantly correlates with a large number of genes that are associated with poor colorectal cancer prognosis (43). (ORM1) has been shown to be upregulated in the serum of breast cancer patients (44); and elevated urinary ORM1 has been suggested as a useful biomarker for bladder cancer (45), ORM1 also plays a key role in hepatocellular carcinoma development and may be a potential target for future development of therapeutic agents against HCC (46). Neurotensin receptor-1 (NTS1) is a G-protein coupled receptor that is being studied in various cancers, where neurotensin (NT) where oncogenic effects in tumors growth and metastatic spread (47, 48).The expression of NTS and its receptor holds potential as a predictive and prognostic marker for colorectal cancer in the postoperative selection of adjuvant therapy (49, 50), Our study Our study suggests that NTS could serve as a predictive and prognostic marker for LUSC.

Patients were subsequently scored based on the expression of the modeled genes using LASSO regression analysis, and divided into high-risk and low-risk groups based on the median risk score. We observed a significant difference in the survival between high-risk and low-risk groups, with patients in the high-risk group exhibiting a lower survival rate.

To validate the accuracy of the risk model, we conducted a validation analysis on the validation set. The results indicated that patients in the high-risk group exhibited lower survival rates in the validation set. The Area Under the Curve (AUC) for both the training set and the validation set at 3 years and 5 years exceeded 0.6, indicating that the reliability of the risk model.

We performed a correlation analysis between risk scores and various clinical traits. The analysis revealed significant differences risk scores across different Ostatus, N stage, and gender subgroups, whereas others clinical traits did not show significant differences (P<0.05). Additionally, we investigated the stratified survival analysis of m7G immune-related gene risk model across clinical traits. The results showed significant differences in survival outcomes for other clinical features except for patients with clinical stage T1, stage III-IV, patients aged ≤ 60 years, females, and non-smokers. Notably, patients aged of ≤ 60 years old had significantly lower risk scores compared to those patients aged >60 years, indicating a better prognosis for the younger cohort.

We examined the expression levels of FGA, CSF3R, NTS, and ORM1 in clinical samples of LUSC. Our findings revealed no significant correlation between the expression of these genes and patients’ gender, age, smoking status, N stage, T stage, or overall clinical stage. Survival analysis indicates that CSF3R, ORM1, and FGA are associated with a poorer prognosis in LUSC patients, whereas NTS is associated with a better prognosis. Based on the expression levels of FGA, CSF3R, NTS, and ORM1, patients were stratified into high-risk and low-risk groups. The high-risk group was associated with a poorer prognosis. The results from clinical samples affirm the robust prognostic capability of the newly developed risk model for LUSC patients.

We utilized clinical factors to construct nomograms that demonstrated the highest AUC values at 1 and 3 years, indicating superior prognostic modeling capacity. Followed this, GSEA were performed. The results indicated that activated in 23 signaling pathways in the high-risk group, including coagulation, E2F targets, G2M checkpoints, IL6/JAK/STAT3 signaling, DNA repair, myogenesis, estrogen response, peroxisomes, angiogenesis, TGF-β signaling, MYC target V1, etc. in the low-risk group, nine pathways were activated, including protein secretion, PI3K/AKT/mTOR signaling, response to interferon alpha, and mitotic spindle. Pathways with a high risk of involvement are associated with poorer LUSC prognosis. Moreover, the higher expression of most immune checkpoint-related genes suggests that patients in high-risk groups may benefit from immunotherapy. We also screened chemotherapeutic agents and small molecules that are sensitive to different risk groups. Notably, the high-risk group exhibited greater sensitivity to commonly used chemotherapeutic agents, such as Lapatinib and Gefitinib. To the best of our knowledge, this represents the first bioinformatics analysis aimed at elucidating the prognostic significance of m7G immune-related gene markers in malignant tumors. However, several limitations warrant considered when interpreting our results. First, our study is based on a retrospective analysis of three public datasets, and our validation in clinical specimens is limited to a single hospital. Therefore, further large-scale and prospective studies are needed for validation. Secondly, unlike m6A modification, the biological processes involving m7G modification remain less comprehensively understood. Therefore, the m7G immune-related genes associated with m7G identified in our study may not encompass the entirety of m7G modification processes. Thirdly, additional detailed mechanistic studies are necessary to elucidate the specific roles of m7G immune-related genes in the development and progression of LUSC. Fourth, this study has some limitations. Specifically, when analyzing sequencing data related to lung squamous cell carcinoma from the TCGA database, only 49 normal lung tissue samples were available. The relatively small sample size may affect the reliability and accuracy of the models we constructed.

In conclusion, we have comprehensively summarized the alterations and prognostic roles of m7G-immune related regulatory genes in LUSC for the first time. We subsequently developed a prognostic model based on the m7G gene signature involving four genes. This model demonstrated robust accuracy in predicting the survival of patients with LUSC and holds potential for guiding personalized treatment decisions. Furthermore, our findings suggest that immune cell infiltration and alterations in the TME may contribute as underlying mechanisms by which the model predicts prognosis in LUSC patients.

## 5 Research highlights

The risk model developed based on m7G immune-related genes has good predictive power, which is helpful in predicting the clinical prognosis of patients with LUSC and guiding treatment decisions.

## Data Availability

The datasets presented in this study can be found in online repositories. The names of the repository/repositories and accession number(s) can be found in the article/**Supplementary Material**.
