# Supplementary material for: Prognostic analysis and identification of M7G immune-related genes in lung squamous cell carcinoma
Source: Front Immunol. 2025 Mar 3;16:1515838. doi: 10.3389/fimmu.2025.1515838 (PMC11911325; doi:10.3389/fimmu.2025.1515838)
Supplement: Supplementary file 1 [file DataSheet1.docx]

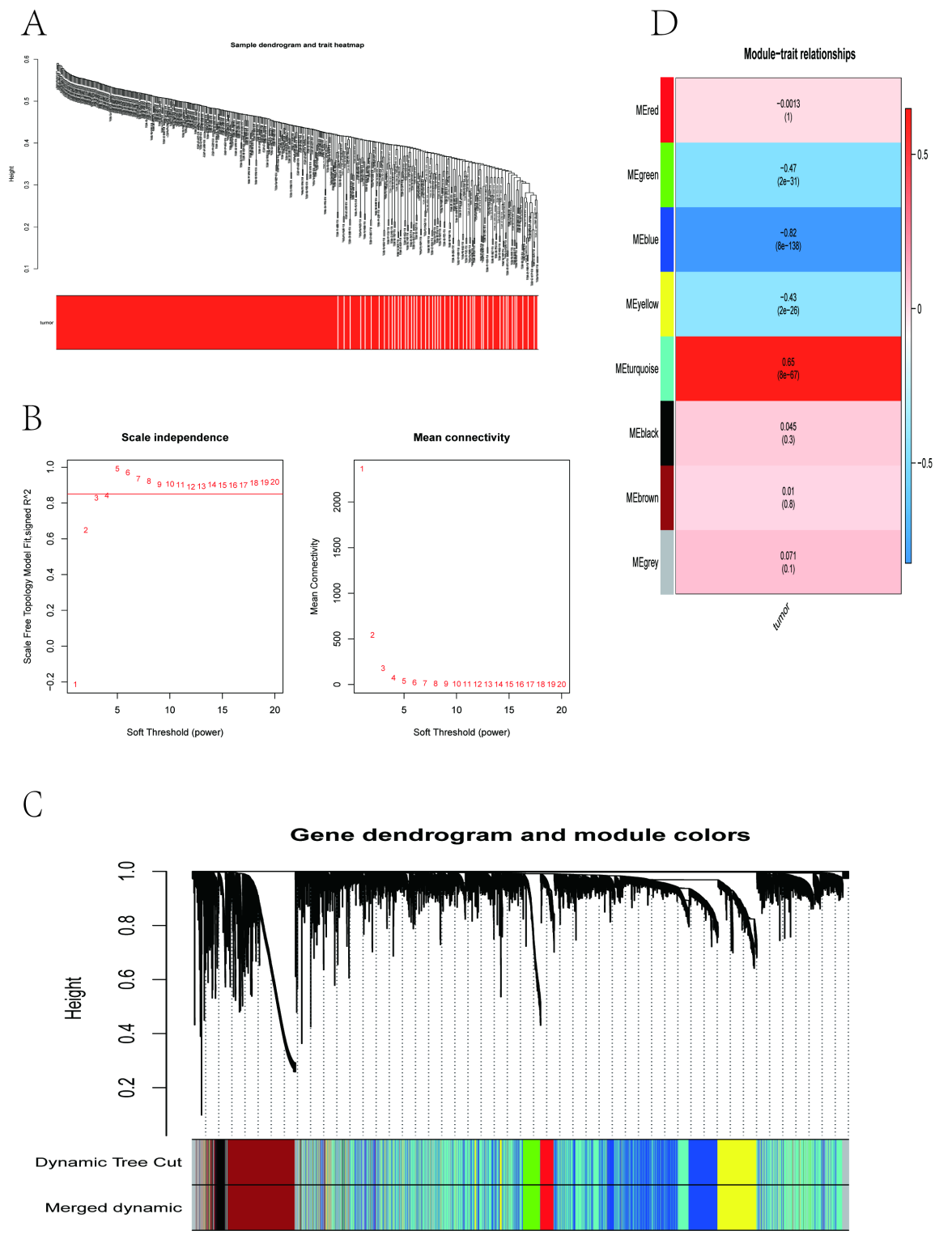


FIGURE S1

LUSC RNA-seq data WGCNA analysis. (A) Identification of a tree of genes associated with clinical information in LUSC. (B) Determination of the soft threshold: When the soft threshold is equal to 5, R² is close to the threshold value of 0.85 (red line), while the mean connectivity on the right is also close to 0. (C) Differential genes were divided into various modules through hierarchical clustering, with different colors representing different modules. (D) Disease-related module genes were grouped, and MEblue and MEturquoise showed the highest correlation with clinical traits, reaching a significant level (p < 0.05).


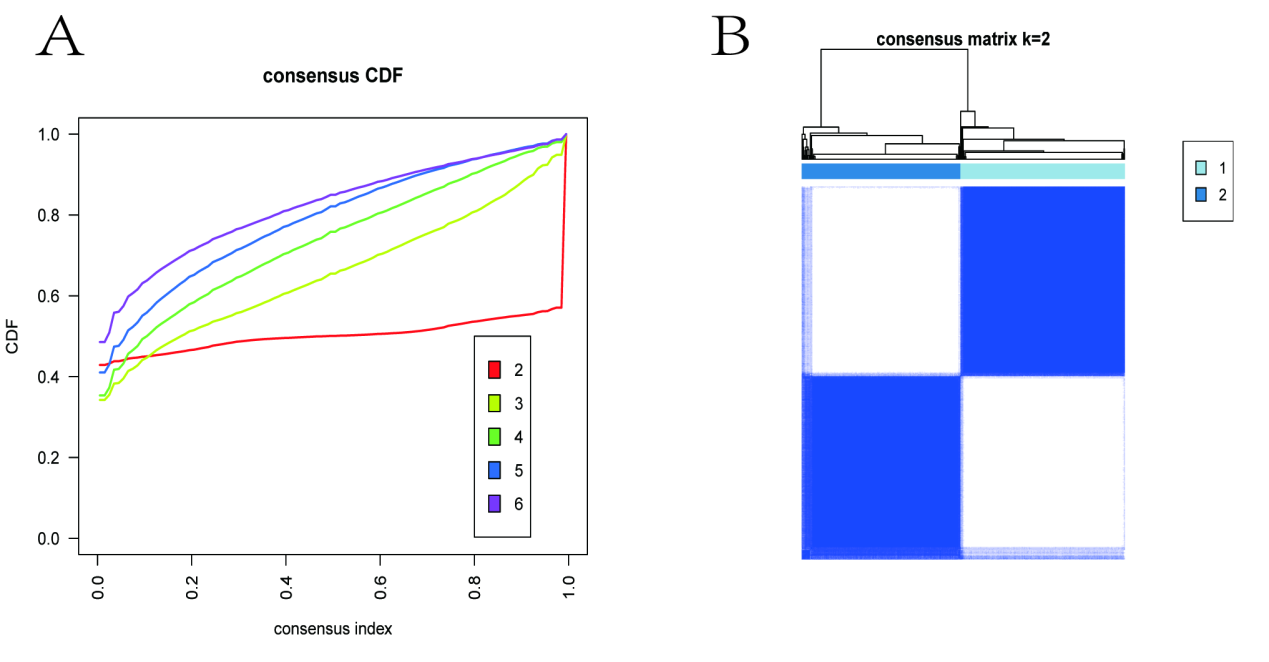


FIGURE S2 Clinical trait analysis of m7G-Associated LUSC subtypes

AB Consensus clustering was used to divide the LUSC patients into 2 clusters (k = 2).
